# The relationship of adverse childhood experiences, hair cortisol, C-reactive protein, and polygenic susceptibility with older adults’ psychological distress during the COVID-19 pandemic

**DOI:** 10.1038/s41380-022-01805-2

**Published:** 2022-10-05

**Authors:** Katie S. Taylor, Andrew Steptoe, Eleonora Iob

**Affiliations:** Department of Behavioural Science and Health, University College London, 1-19 Torrington Place, WC1E 7HB London, UK

**Keywords:** Depression, Predictive markers, Psychology, Genetics, Biochemistry

## Abstract

Adverse childhood experiences (ACEs) are linked to poorer mental health outcomes, and growing evidence implicates biological and genetic pathways from early adversity to psychopathology. However, little is known about the relationship of ACEs and their underlying biological and genetic mechanisms with older people’s mental health responses to the COVID-19 pandemic. We tested the associations of ACEs, hair cortisol, C-reactive protein (CRP), and polygenic scores (PGS) with depression, anxiety, and loneliness among older adults during the COVID-19 pandemic, accounting for the potential interplay of ACEs with biological and genetic risk markers. Data were drawn from the English Longitudinal Study of Ageing, a prospective cohort study of older adults living in England. Retrospective information on ACEs were collected in 2006/7, while CRP and hair cortisol were measured at wave 6 (2012/13). Psychological distress was assessed before the pandemic (2018–19) and at two COVID-19 assessments in 2020 (June-July and November-December). Associations were tested on 2050 participants using linear/logistic regression models adjusted for pre-pandemic outcome measures and mixed-effect models to assess changes before and during the pandemic. The results showed that ACEs were associated with higher levels of depression (OR = 2.55[95%CI:1.81,3.59]) anxiety (OR = 1.84[95%CI:1.13,3.01]), and loneliness (b = 0.28[95%CI:0.14,0.42]) during the pandemic. Hair cortisol was related to an increased risk of depression (OR = 1.15[95%CI:1.04,1.26]), and CRP was associated with greater loneliness scores (b = 0.16[95%CI:0.03,0.30]). The relationship between cortisol and psychological distress was larger among participants with ACEs (*e.g*., OR_depression_ = 1.07[95%CI:1.00,1.14]). Further, individuals with high CRP experienced greater increases in feelings of loneliness from before to during the pandemic, compared to those with lower CRP levels (interaction effect=0.23; 95%CI:0.1–0.37). Individuals with 2+ ACEs experienced greater increases in depressive symptoms compared to those with none (interaction effect=2.09; 95%CI:1.1–3.98). Higher levels of hair cortisol were also related to worse changes in depressive symptoms across timepoints (interaction effect=1.84;95%CI:1.41–2.41). These results highlight the lasting impact of biosocial vulnerabilities on older adults’ mental health responses to new environmental stressors. They also implicate biological mechanisms in the pathophysiology of later-life psychological distress.

## Introduction

The COVID-19 pandemic is associated with adverse consequences for many aspects of people’s lives, including physical and mental health, employment and financial security, social relationships, and access to health and social care services [1]. Given a stress-diathesis framework, the pandemic can be seen as a new source of chronic environmental stressors accentuating the negative impact of pre-existing social, biological, or genetic vulnerabilities on mental health [2]. Older adults have been at increased risk of social isolation throughout the pandemic as they are particularly vulnerable to severe illness following infection [3]. Indeed, the pandemic has been linked to both immediate and longer-term adverse mental health consequences among this population, including increases in depression, anxiety, loneliness, and poor quality of life [4]. However, little is known about the impact of the COVID-19 crisis as an environmental stressor on older adults’ mental health responses among those with greater social, biological, or genetic vulnerability.

Adverse childhood experiences (ACEs), such as abuse and neglect [5], are consistently associated with poor mental health in later-life [6] and during the pandemic [7]. Environmental stress may play a role in these associations between ACEs and mental health during the pandemic, which supports a stress-diathesis framework for pandemic psychological distress [2]. The long-lasting effects of ACEs on mental health may be explained by dysregulated levels of inflammation and altered hypothalamic-pituitary-adrenal (HPA)-axis activity [8, 9]. Impairments to these biological systems are associated with heightened stress reactivity [10], which may explain exaggerated reactions to new environmental stressors [11], such as the pandemic. Furthermore, dysregulated cortisol and inflammation levels are associated with poorer mental health outcomes, including depression and anxiety [12–14], hence indicating a biological pathway from ACEs to psychopathology [15].

The interplay of ACEs with HPA-axis function and inflammation is supported by research observing dysregulated cortisol levels and elevated CRP in individuals who experienced early-life adversity [16]. However, the precise nature of these associations is less clear. For example, research has identified both hypoactivation and hyperactivation of the HPA-axis following exposure to ACEs [17]. Such inconsistencies may be explained in part by the reliance on cortisol measurements in body fluids (e.g., saliva, blood, urine). These measures represent short-term cortisol levels and are influenced by interindividual and environmental fluctuations [18]. Hair cortisol, however, is considered a reliable measure of longer-term cortisol exposure [19]. Whilst hair cortisol is a more appropriate method to study the long-term impacts of ACEs on HPA-axis function, the findings using hair cortisol are generally still mixed [20]. Regarding inflammation, a consistent association between ACEs and elevated inflammatory markers is observed in adults [8, 21], but not children [22–24]. Furthermore, threat-related adversities (e.g. physical abuse) are more strongly related to adverse stress-related biological outcomes than others (e.g. deprivation) [8, 23, 25]. This suggests that both the type and timing of adversity are important in understanding the relationship between ACEs and adverse biological outcomes [20].

Genetic factors are also shown to influence psychological distress [26]. Results from genome-wide association studies (GWAS) suggest that mental health disorders are complex polygenic traits, meaning their genetic influences involve thousands of DNA variants that each have very small influences [27]. Polygenic scores (PGS) reflect an individual’s inherited susceptibility to a disease, calculated by summing the risk alleles carried by an individual [28]. As PGS consider the polygenic nature of mental health disorders, they are an appropriate method for assessing the complex genetic basis of psychiatric disorders [29, 30] and observations demonstrate associations between specific PGS and psychological distress [31]. Furthermore, the negative impact of ACEs is accentuated among certain individuals due to their genetic architecture [32]. The interplay between ACEs and PGS for depression and inflammation has been shown to increase the risk of depression and inflammation beyond their individual main effects [33], thereby providing evidence of the gene-environment mechanisms involved in psychopathology development.

Investigating the role of ACEs and their underlying biological and gene-environment pathways in psychological distress is particularly relevant to older adults since ageing is associated with increased cortisol levels, upregulated inflammatory responses, increased risk of cognitive decline, and reduced social connections [34]. Moreover, it is unclear whether these risk factors and their interactions may also affect older people’s psychological reactions to new environmental stressors such as the pandemic. A clearer understanding of pre-existing vulnerabilities associated with worse mental health following the exposure to new environmental stressors would help to identify vulnerable individuals and develop novel interventions targeting mechanistic pathways.

**Fig. 1:**
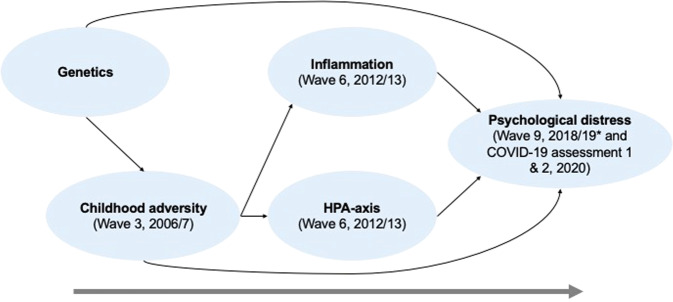
The proposed theoretical framework. **Note**. Arrows represent the relationships explored in the current study. The assessment points and waves the measures were taken, with their relevant years, are detailed in brackets. The large arrow at the bottom of the diagram represents time. The * is to show that the pre-COVID-19 psychological distress measures were not the outcomes in the current study but were included in some analyses.

The overall aim of the current study was to investigate the individual differences in older adults’ psychological response to the pandemic. Specifically, we tested whether ACEs, hair cortisol, CRP, and PGS are independent risk factors for depression, anxiety, and loneliness among older adults during the COVID-19 pandemic, and we also examined the associations of these risk factors with changes in psychological distress before and during the pandemic. We took account of any associations between these factors and pre-pandemic distress, to ensure that our findings were not secondary to existing relationships. Furthermore, the interactions of ACEs with cortisol, CRP, and PGS were tested to understand the gene-environment and biology-environment interactions influencing psychological distress during the pandemic. We hypothesised that having ACEs, higher levels of cortisol and inflammation, and higher PGS would be associated with greater psychological distress during the pandemic and greater worsening of mental health from before to during the pandemic. We also hypothesised that ACEs would interact with inflammation, cortisol, and PGS to further increase an individual’s risk of psychological distress. Figure 1 represents the theoretical framework for the study.

## Materials and methods

### Sample

Data from the English Longitudinal Study of Ageing (ELSA) were used in the current study. ELSA is a prospective population-based cohort study of adults living in England, aged 50 years and older [35]. In 2020, a COVID-19 sub-study based on the regular ELSA sample was initiated to assess psychosocial experiences during the pandemic [36]. For this study, depression, anxiety, and loneliness were ascertained at the two COVID-19 sub-study assessments in June-July and November-December 2020 and at the most recent regular ELSA wave before the pandemic – i.e., wave 9 (2018/2019). During the third regular wave of the study (2006/2007), ACEs were retrospectively assessed as part of a life history interview. CRP and hair cortisol measurements were collected in wave 6 (2012/2013) during the nurse visits. Included in the current analyses were core ELSA members who took part in both COVID-19 sub-study assessments and in wave 9, completed the life history interview at wave 3, and with valid cortisol and CRP measurements and genetic data (*N* = 2050). All respondents provided informed consent. Ethical approval for the regular ELSA study was obtained from the National Research Ethics Service. The ELSA COVID-19 Substudy has been approved by the University College London Research Ethics Committee.

### Measures

#### Outcomes

##### Depression

Depressive symptoms were assessed with the 8-item Centre for Epidemiological Studies Depression (CESD-8) scale. This measure is validated for the assessment of depression among older adults [37, 38] and has been shown to have good internal consistency at each wave of ELSA (Cronbach’s a > 0.95) [39]. One item (*i.e*., “felt sad much of the time”) was unintentionally omitted from the COVID-19 sub-study. Therefore, a seven-item scale was used for both the COVID-19 assessments and wave 9. At each assessment a total score was calculated (range: 0–7), representing the total number of depressive symptoms a participant reported. A binary score was created with ≥4 symptoms indicating likely cases of clinical depression [4]. This cut-off has been validated for the 8-item CESD scale against standardised psychiatric interviews in older populations [40] but was not adjusted for the 7-item scale used in the current study.

##### Anxiety

The 7-item Generalised Anxiety Disorder scale (GAD-7) was used to measure symptoms of anxiety at both COVID-19 assessments. At each assessment a total score was calculated (range: 0–21), representing the total number of anxiety symptoms a participant reported. This measure is validated for the assessment of anxiety in the older population [41] and has been shown to have good internal consistency (Cronbach’s a 0.79–0.91) [42]. A total score of 10 or greater was used as a cut-off point for identifying likely cases of GAD [43]. The GAD-7 was not administered in the regular ELSA waves; to take account of anxiety levels pre-pandemic we used the Office for National Statistics anxiety scale administered in wave 9 [44].

##### Loneliness

The 3-item revised University of California (UCLA) Loneliness scale and an additional item asking participants how often they feel lonely were used to measure loneliness levels at wave 9 and both COVID-19 assessments. This measure has been validated among older adults [45] and has been shown to have high internal consistency within ELSA (Cronbach’s a = 0.83) [46]. At each assessment the item scores were summed to derive a total loneliness score, with higher values indicating greater loneliness (range: 1–12).

#### Exposures

##### Adverse childhood experiences

All ACE data came from the life history interview during the third regular ELSA wave (2006/2007). This interview aimed to collect retrospective information about the early life experiences of participants. This data has been validated against prospective data from the National Child Development Study [47]. Numerous studies have used ACEs data from ELSA to assess their associations with various adulthood outcomes [48, 49]. A recent meta-analysis questioned the agreement between prospective and retrospective measures of childhood maltreatment, concluding the two measures cannot be used interchangeably [50]. Therefore, it is important to acknowledge these critical measurement differences.

Informed by the ACEs definition provided by McLaughlin and colleagues [51], items that likely require significant adaptation from a child and might be instrumental in predicting long-term health outcomes were selected. Up to the age of 16, 12 different types of ACEs were considered, including sexual abuse, physical assault, physical abuse from parents, parent arguments, parent mental illness or substance abuse, parent separation or divorce, poor maternal and paternal bonding, separation from mother for more than six months, parent death, foster care or adoption, and institutionalisation. Frequencies of the sample for each type of ACE are detailed in Supplementary Table 1. Previous research has identified distinct dimensions of ACEs in the ELSA sample, namely Threat, Household Dysfunction, Bonding, and Loss experiences [8], displayed in the Appendix, Supplementary Table 2. Parental bonding was assessed using the 7-item Parental Bonding Instrument (PBI) [52], designed to retrospectively assess adults’ perceptions of their parents’ parenting styles. Total bonding scores were calculated separately for each parent and ranged from 0 (highest bonding) to 7 (lowest bonding). Two binary measures of low maternal/paternal bonding were derived using a total score >3, an approach used in previous research [8]. For the remaining items, participants reported whether they had ever experienced that event during childhood. For the main analyses, a cumulative risk score indicating the total number of ACEs reported was used, including the two binary parental bonding scores. This variable was then categorised into experiencing no ACEs (*N* = 1060), 1 ACE (*N* = 458), or multiple ACEs (≥2) (*N* = 532). For a sensitivity analysis, dichotomised scores representing the presence or absence of at least one type of ACE included in each dimension were derived.

##### C-reactive protein

Blood samples for the CRP assessment were collected from participants at wave 6 by study nurses in their own homes. High sensitivity CRP plasma concentrations were assayed using the N latex CRP mono Immunoassay on the Behring Nephelometer II Analyser. For further details on these methods in ELSA, see Supplementary Materials. A binary score was created indicating lower and higher CRP, using a commonly accepted threshold (≥3 mg/l) to indicate clinically significant elevated CRP levels [53].

##### Hair cortisol

During wave 6, hair strands of ~3 cm, weighing at least 10 mg were collected as close to the scalp as possible from the posterior vertex. The 3 cm hair sample closest to the scalp is presumed to supply a measure of the average cortisol output over the three preceding months, based on the average ~1 cm hair growth per month [54]. Exclusion criteria for hair sampling included pregnancy, breastfeeding, certain scalp conditions, inability to sit with head remaining still, and having less than 2 cm of hair length in the required area. Hair analysis was conducted by the Technische Universität Dresden (Germany). Cortisol levels were quantified by high performance liquid chromatography-mass spectrometry following a standard wash and steroid extraction procedure [55], expressed in pg/mg. Considering that hair cortisol and CRP were measured 8 years before the pandemic, these measures most likely reflect the accumulation of biopsychosocial and behavioural exposures across time, including the long-term impact of ACEs as demonstrated by earlier research in ELSA [8].

##### Polygenic scores

PGS of major depressive disorder [56] (MDD-PGS), anxiety [57] (ANXIETY-PGS), and loneliness [58] (LONE-PGS) were constructed using summary statistics from large GWAS meta-analyses with PLINK and PRSice software. A single *P*-value threshold of 1 for all PGS was used as PGSs including all available single-nucleotide polymorphism (SNPs) have been shown to either explain the most amount of variation in a trait or are not significantly different than PGSs based on different *P*-value thresholds [33]. This maximised the potential predictive ability of the PGSs and reduced multiple testing. Further details regarding the derivation of PGS in ELSA can be found in the relevant documentation report [59] and in Supplementary Materials.

#### Covariates

Based on previous research, statistical analyses were adjusted for covariates. These included adult sociodemographic factors (age, gender, partnership status, living alone, wealth tertiles), use of medication (anti-inflammatory/antihypertensive drugs, steroids), childhood socioeconomic factors (overcrowding, number of books in the home, financial hardship, parent unemployment), hair characteristics (dyed, colour, season of collection, phase of hair analysis), and genetic population stratification by including 10 principal components. The analyses were adjusted for pre-pandemic psychological distress scores (depression, anxiety, and loneliness at wave 9) to isolate the specific impact of the pandemic on psychological distress. Lifestyle factors (physical activity, alcohol consumption, smoking status, and BMI), limiting long-standing illnesses, and COVID-19-related worries (financial insecurity, severe risk of illness following infection, friend or family member died from coronavirus, COVID-19 infection) were included in sensitivity analyses.

### Statistical analyses

All statistical analyses were conducted in R Studio version 2022.02.3. The code of the statistical analyses is available from the corresponding author upon request.

#### Main analyses

Descriptive statistics are reported as means (M) and standard deviations (SD) or *n* (%). The cortisol measure was log transformed as the distribution was positively skewed. An average COVID-19 score across the two COVID-19 assessments was calculated for each outcome to provide a single value of pandemic psychological distress. Multiple logistic (depression and anxiety) and linear (loneliness) regression analyses were first conducted for individual associations between each exposure and outcome measure. The first models for each outcome assessed their individual associations with ACEs, adjusting for adult sociodemographic factors, childhood socioeconomic position, and pre-pandemic outcome score. The second models assessed associations with cortisol, adjusting for adult sociodemographic factors, use of steroids, hair characteristics, and pre-pandemic outcome score. The third models assessed associations with CRP, adjusting for adult sociodemographic factors, use of anti-inflammatory/anti-hypertensive medication, and pre-pandemic outcome score. The final models assessed associations for each outcome with their respective PGS (e.g., MDD-PGS for depression), adjusting for principal components for ancestry and pre-pandemic outcome score.

Multiple logistic and linear regression analyses were then conducted for mutually adjusted associations and interactions for each outcome measure, meaning all exposure variables were included in the models (ACEs, cortisol, CRP, and PGS). To improve power and interpretation, the continuous ACEs variable was used in all mutually adjusted and interaction models. The first models assessed the association of ACEs, cortisol, CRP and PGS with the individual psychological outcomes, adjusting for all covariates included in the individual models. The models for each outcome were the same as the first model with the inclusion of interaction terms between ACEs and either cortisol, CRP, or PGS.

Mixed-effect models, outlined in Supplementary Materials, were subsequently used to examine changes in psychological distress during the COVID-19 pandemic. A linear model was conducted to estimate changes in loneliness score and a logistic model was conducted to estimate changes in depression. For anxiety, no mixed-effect models were conducted as the pre-pandemic measure differed from that used in the COVID-19 assessments. Changes in loneliness and depression were estimated using a binary predictor variable that indicated whether the outcome was measured before or during the COVID-19 pandemic. Interaction effects between the COVID-19 period indicator and the 4 exposures (ACEs, hair cortisol, CRP, and PGS) were examined to understand whether and how the change in psychological distress outcomes might vary across different pre-existing biosocial vulnerabilities.

#### Sensitivity analyses

Lifestyle factors and limiting long-standing illness were included as additional covariates in the mutually adjusted regression models. These variables were not included in the main analyses as they could be intermediate variables on the causal pathways leading from ACEs to psychological distress. The individual associations of distinct ACE dimensions (threat, household dysfunction, bonding, and loss experiences) with each outcome were assessed. This was used to identify whether certain dimensions were more strongly related to psychological distress than others. Regression analyses were repeated with the exclusion of participants with CRP levels >10 mg/L, as this may indicate the presence of a current infection rather than chronic low-grade inflammation. Regression analyses were repeated with a continuous CRP measure to determine whether categorisation impacted the results. The analyses were repeated with wave 9 psychological distress measures as the outcome variables to understand whether the exposures were associated with the outcomes independently from the pandemic. The mutually adjusted regression models were repeated with continuous anxiety and depression scores to see whether dichotomisation impacted the results. Mutually adjusted regression models were also repeated with the inclusion of variables representing COVID-19-related worries as additional covariates to understand the degree of impact the pandemic had on individuals. Due to multiple comparisons, *p*-values were adjusted using the Benjamini-Hochberg procedure to decrease false discovery rate. Lastly, subgroup analyses were conducted to understand whether the participants excluded from the current study differed meaningfully from those included.

## Results

### Descriptives

Participant characteristics are displayed in Table 1. Participants (*N* = 2 050) had a mean age of 75.15 (±6.50; *range* = 64–99), with 34% male and 66% female. Most participants (51.7%) had experienced no ACEs, whilst 22.3% and 26.0% experienced 1 and 2+ ACEs, respectively. Participants had mean cortisol (log) levels of ~2.13 (±1.50; *range* = −0.73–8.79) and 24.7% had high CRP. These levels are comparable to other samples of similar ages [60, 61]. Before the pandemic, 7.7% of participants had clinically significant depressive symptoms compared with 15.1% and 17.2% at the first and second COVID-19 assessments, respectively. The number of participants with clinically significant anxiety symptoms were 6.1% and 7.1% at the first and second COVID-19 assessments, respectively. Mean loneliness pre-pandemic was ~5.16 (±1.84; *range* = 1–12) compared to ~5.45 (±1.91; *range* = 3–12) and ~5.51 (±1.98; *range* = 4–12) at the first and second COVID-19 assessments, respectively. These data suggest an increase in psychological distress during the pandemic, as reported elsewhere [4].

**Table 1:** Sample characteristics.

Variable	Overall (*N* = 2050)
Sociodemographic characteristics (COVID-19 w1)	
*Age*	
Mean (SD)	75.15 (6.51)
Range	64.00–99.00
*Sex*	
Males	702 (34.2%)
Females	1 348 (65.8%)
*Living alone*	
Missing	6 (0.3%)
No	1432 (70.1%)
Yes	612 (29.9%)
*Has partner*	
Yes	1348 (65.8%)
No	702 (34.2%)
*Wealth tertiles*	
Missing	4 (0.2%)
High	682 (33.3%)
Medium	682 (33.3%)
Low	682 (33.3%)
*Limiting long-standing illness*	
Yes	1158 (56.5%)
No	892 (43.5%)
*Early-life experiences (Life history interview, w3)*	
Adverse Childhood Experiences	
0	1060 (51.7%)
1	458 (22.3%)
2+	532 (26.0%)
*Childhood socioeconomic adversities*	
Missing	12 (0.6%)
Mean (SD)	0.46 (0.72)
Range	0–4
*Stress-related biomarkers, medications, and lifestyle factors (w6)*	
*C-Reactive protein*	
Not high CRP	1544 (75.3%)
High CRP	506 (24.7%)
*Hair cortisol (log)*	
Mean (SD)	2.13 (1.50)
Range	−0.73–8.79
*Anti-inflammatory medication*	
No	1140 (55.6%)
Yes	910 (44.4%)
*Steroids*	
No	1976 (96.4%)
Yes	74 (3.6%)
*BMI*	
Missing	30 (1.5%)
Mean (SD)	28.02 (4.96)
Range	15.69–50.36
*Smoking status*	
Not a current smoker	1906 (93.0%)
Current smoker	144 (7.0%)
*Alcohol consumption*^1^	
Missing	72 (3.5%)
Mean (SD)	4.88 (2.12)
Range	1.0–8.0
*Physically active*	
No	1090 (53.2%)
Yes	960 (46.8%)
*Mental health (COVID-19 average)*	
*Elevated depressive symptoms (CESD-8* *=* *>4)*	
No	1788 (87.2%)
Yes	262 (12.8%)
*Anxiety (Gad-7* *=* *>10)*	
No	1936 (94.4%)
Yes	114 (5.6%)
*Loneliness*	
Mean (SD)	5.481 (1.804)
Range	3.5–12.0

^1^Higher values in the alcohol consumption variable indicate lower consumption.

### Individual associations

The individual regression models for depression, anxiety, and loneliness are summarised in Table 2. ACEs were positively associated with all psychological distress outcomes during the pandemic. If a participant had 1 or 2+ ACEs, the odds of depression were 1.82 (95%CI:1.24;2.65) and 2.55 (95%CI:1.81;3.59) times greater than if they had no ACEs, respectively. Similarly, having ACEs was positively associated with anxiety. If a participant had 1 or 2+ ACEs, the odds of anxiety were 1.79 (95%CI:1.04;3.05) and 1.84 (95%CI:1.13;3.01) times greater than if they had no ACEs, respectively. Finally, ACEs were positively related to loneliness, such that having 2+ ACEs (versus none) was associated with an average increase of 0.28 (95%CI:0.14;0.42) in the total loneliness score. Hair cortisol was also associated with depressive symptoms, such that for every one-unit increase in hair cortisol (log), the odds of having depressive symptoms increased by 1.15 (95%CI:1.04;1.26) times. CRP was associated with loneliness, such that predicted loneliness scores for an individual with high CRP was 0.16 (95%CI:0.03;0.30) times greater than that predicted for participants without high CRP.

**Table 2:** Regression models for the individual and mutually adjusted associations of ACEs, hair cortisol, CRP, and PGS with depression, anxiety, and loneliness.

Term	Model^1^	Estimate (OR/b)	Standard error	*P* value	FDR adjusted *p*-value	Confidence low	Confidence high
*Depression*^2,3^							
1 ACE^4^	1	1.817	0.193	0.002	0.014	1.242	2.646
2+ ACEs^4^	1	2.546	0.175	<0.001	<0.001	1.808	3.593
Total ACEs^5^	2	1.223	0.050	<0.001	0.001	1.108	1.348
Hair cortisol	1	1.145	0.048	0.005	0.031	1.041	1.256
Hair cortisol	2	1.143	0.050	0.008	0.040	1.034	1.260
High CRP^4^	1	1.091	0.164	0.597	0.785	0.787	1.499
High CRP^4^	2	1.045	0.178	0.807	0.867	0.733	1.474
MDD PGS^6^	1	1.101	0.075	0.199	0.471	0.951	1.277
MDD PGS^6^	2	1.065	0.080	0.436	0.664	0.910	1.248
*Anxiety*^2.3^							
1 ACE^4^	1	1.787	0.274	0.034	0.128	1.037	3.053
2+ ACEs^4^	1	1.841	0.249	0.014	0.064	1.131	3.008
Total ACEs^5^	2	1.141	0.069	0.054	0.170	0.993	1.300
Hair cortisol	1	1.095	0.072	0.209	0.471	0.947	1.256
Hair cortisol	2	1.083	0.076	0.293	0.577	0.930	1.253
High CRP^4^	1	1.239	0.228	0.348	0.626	0.785	1.921
High CRP^4^	2	1.130	0.248	0.622	0.785	0.688	1.825
ANX PGS^6^	1	0.950	0.104	0.621	0.785	0.774	1.165
ANX PGS^6^	2	0.907	0.113	0.387	0.634	0.726	1.132
*Loneliness*^3^							
1 ACE^4^	1	−0.02	0.074	0.786	0.857	−0.166	0.125
2+ ACEs^4^	1	0.279	0.072	<0.001	<0.001	0.138	0.421
Total ACEs^5^	2	0.094	0.022	<0.001	<0.001	0.051	0.137
Hair cortisol	1	−0.001	0.020	0.963	0.963	−0.040	0.038
Hair cortisol	2	−0.009	0.020	0.666	0.799	−0.047	0.030
High CRP^4^	1	0.163	0.069	0.019	0.080	0.027	0.299
High CRP^4^	2	0.149	0.071	0.035	0.128	0.010	0.288
LONE PGS^6^	1	−0.011	0.030	0.713	0.818	−0.070	0.048
LONE PGS^6^	2	−0.032	0.030	0.279	0.577	−0.091	0.026

^1^ Model 1 refers to the individual associations and model 2 refers to the mutually adjusted associations between exposure and outcome.

^2^Odds ratios are presented for anxiety and depression.

^3^Covariates included in the models: pre-pandemic psychological distress (depression, anxiety, and loneliness respectively), adult sociodemographic factors (age, gender, partnership status, living alone, wealth tertiles), use of medication (anti-inflammatory/antihypertensive drugs, steroids), childhood socioeconomic factors (overcrowding, number of books in home, financial hardship, parent unemployment), hair characteristics (dyed, colour, season of collection, phase of hair analysis), and genetic population stratification by including 10 principal components.

^4^Having 0 ACEs and not having high CRP were the reference categories.

^5^The continuous ACEs variable was used for all mutually adjusted regression models, i.e., model 2.

^6^MDD PGS, ANX PGS, and LONE PGS are the PGS for depression, anxiety, and loneliness, respectively.

### Mutually adjusted associations and interaction effects

The mutually adjusted regression models for loneliness, depression, and anxiety are summarised in Table 2. Following the adjustment for all exposures, ACEs and hair cortisol remained associated with depression, such that for every additional ACE and one-unit increase in cortisol, the odds of having depressive symptoms increased by 1.22 (95%CI:1.11;1.35) and 1.14 (95%CI:1.03;1.26) times, respectively. ACEs and CRP remained associated with loneliness, such that every additional ACE was associated with an increase in total loneliness score by 0.09 points (95%CI:0.05;0.14). Likewise, predicted loneliness score for individuals with high CRP was 0.15 (95%CI:0.01;0.29) times greater than for participants without high CRP. ACEs were no longer associated with anxiety when accounting for all exposures.

**Fig. 2:**
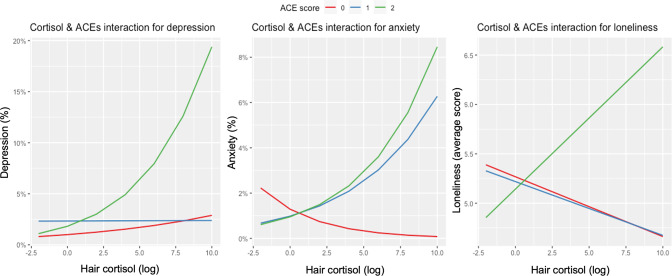
Interaction plot between ACEs and hair cortisol for depression, anxiety, and loneliness. **Note**. Sample: ELSA (*N* = 2050). Covariates included in the models: pre-pandemic psychological distress (depression, anxiety, and loneliness), adult sociodemographic factors (age, gender, partnership status, living alone, wealth tertiles), use of medication (anti-inflammatory/antihypertensive drugs, steroids), childhood socioeconomic factors (overcrowding, number of books in home, financial hardship, parent unemployment), hair characteristics (dyed, colour, season of collection, phase of hair analysis), and genetic population stratification by including 10 principal components.

Interaction effects between ACEs and hair cortisol were observed for all psychological distress outcomes and are illustrated in Table 3 and Fig. 2, for full results see Appendix, Supplementary Table 3. These interaction effects indicate that the relationship of hair cortisol with depression, anxiety, and loneliness was larger among participants with ACEs than in those without (OR_depression_ = 1.07, 95%CI:[1.00;1.14]; OR_anxiety_ = 1.17, 95%CI:[1.06;1.28]; b_loneliness_ = 0.04, 95%CI:[0.01;0.07]). To illustrate, for every 1-unit increase in hair cortisol, the odds of depression were 1.06 times greater for individuals with no ACEs, compared to 1.48 greater odds of depression for individuals with 5 ACEs. For every 1-unit increase in hair cortisol, the odds of anxiety decreased by 13% for individuals with no ACEs, whereas they were 1.88 times greater for individuals with 5 ACEs. Finally, predicted loneliness score for individuals with no ACEs decreased by 0.04 points for every 1-unit increase in hair cortisol, whereas it increased by 0.2 points for individuals with 5 ACEs. No interaction effects were observed between ACEs and CRP or PGS for any psychological distress outcomes.

**Table 3:** Regression models of the significant mutually adjusted interactions between ACEs and hair cortisol for depression, anxiety, and loneliness.

Term	Estimate (OR/b)	Standard error	*P* value	FDR adjusted *p*-value	Confidence low	Confidence high
*Depression*^1,2^						
Total ACEs	1.054	0.091	0.566	0.777	0.878	1.258
Hair cortisol	1.060	0.065	0.369	0.633	0.931	1.200
ACEs*Cortisol	1.069	0.034	0.048	0.156	1.001	1.142
*Anxiety*^1,2^						
Total ACEs	0.814	0.134	0.123	0.329	0.621	1.050
Hair cortisol	0.872	0.107	0.203	0.471	0.703	1.071
ACEs*Cortisol	1.166	0.049	0.002	0.012	1.060	1.283
*Loneliness*^2^						
Total ACEs	0.015	0.039	0.701	0.818	−0.061	0.090
Hair cortisol	−0.043	0.024	0.074	0.214	−0.090	0.004
ACEs*Cortisol	0.039	0.015	0.012	0.058	0.008	0.069

^1^Odds ratios are presented for anxiety and depression.

^2^Covariates included in the models: pre-pandemic psychological distress (depression, anxiety, and loneliness), adult sociodemographic factors (age, gender, partnership status, living alone, wealth tertiles), use of medication (anti-inflammatory/antihypertensive drugs, steroids), childhood socioeconomic factors (overcrowding, number of books in home, financial hardship, parent unemployment), hair characteristics (dyed, colour, season of collection, phase of hair analysis), and genetic population stratification by including 10 principal components.

### Mixed-effect models

The mixed-effect models for loneliness and depression are summarised in Table 4. Individuals with high CRP experienced worse changes in loneliness from pre-pandemic to during the pandemic, compared to those with lower CRP scores (interaction effect=0.23; 95%CI:0.1–0.37). Individuals with 2+ ACEs experienced worse changes in depression compared to those without ACEs (interaction effect=2.09; 95%CI:1.1–3.98). Finally, hair cortisol levels were positively associated with worse changes in depression (interaction effect=1.84;95%CI:1.41–2.41) across the timepoints.

**Table 4:** Mixed effect models of the individual effects and interaction effects of COVID-19 period indicator with ACEs, hair cortisol, CRP, and PGS on depression and loneliness.

Term	Estimate (OR/b)	Standard error	*P* value	FDR adjusted *p*-value	Confidence low	Confidence high
*Depression*^1,2^						
COVID-19^3^	4.94	0.138	<0.001	<0.001	3.78	6.47
1 ACE^4^	1.55	0.430	0.311	0.466	0.67	3.60
2+ ACEs^4^	3.79	0.372	<0.001	0.001	1.83	7.85
Cortisol^4^	0.97	0.136	0.849	0.849	0.75	1.27
CRP^4^	1.33	0.443	0.521	0.625	0.56	3.17
MDD PGS^4,5^	1.27	0.222	0.279	0.466	0.82	1.96
COVID*1 ACE	1.31	0.378	0.472	0.529	0.63	2.75
COVID*2+ ACEs	2.09	0.328	0.024	0.049	1.10	3.98
COVID*cortisol	1.84	0.136	<0.001	<0.001	1.41	2.41
COVID*CRP	1.52	0.340	0.216	0.268	0.78	2.97
COVID*MDD PGS^5^	0.78	0.150	0.098	0.161	0.58	1.05
*Loneliness*^2^						
COVID-19^3^	0.33	0.031	<0.001	<0.001	0.27	0.39
1 ACE^4^	0.20	0.090	0.027	0.066	0.02	0.37
2+ ACEs^4^	0.54	0.086	<0.001	<0.001	0.37	0.70
Cortisol^4^	−0.02	0.024	0.428	0.571	−0.07	0.03
CRP^4^	0.02	0.084	0.819	0.849	−0.14	0.18
LONE PGS^4,5^	0.06	0.037	0.114	0.229	−0.01	0.13
COVID*1 ACE	−0.09	0.077	0.220	0.268	−0.25	0.06
COVID*2+ ACEs	0.11	0.074	0.144	0.212	−0.04	0.25
COVID*cortisol	0.01	0.020	0.696	0.725	−0.03	0.05
COVID*CRP	0.23	0.071	0.001	0.003	0.10	0.37
COVID*LONE PGS^5^	−0.04	0.031	0.196	0.262	−0.10	0.02

^1^Odds ratios are presented for depression.

^2^Covariates included in the models: adult sociodemographic factors (age, gender, partnership status, living alone, wealth tertiles), use of medication (anti-inflammatory/antihypertensive drugs, steroids), childhood socioeconomic factors (overcrowding, number of books in home, financial hardship, parent unemployment), hair characteristics (dyed, colour, season of collection, phase of hair analysis), and genetic population stratification by including 10 principal components.

^3^The COVID-19 variable represents the binary variable of whether data was collected before or during the pandemic and is the value taken from mutually adjusted models (i.e., when ACEs, cortisol, CRP, and PGS are included in one model).

^4^For the ACEs, cortisol, CRP, and PGS associations with depression and loneliness, the models presented were the individual models which did not include the interaction effects.

^5^MDD PGS and LONE PGS are the PGS for depression and loneliness, respectively.

### Sensitivity analyses

The inclusion of lifestyle factors and limiting longstanding illness did not alter the associations between ACEs and psychological distress outcomes or hair cortisol and depression. However, they did attenuate the relationship between CRP and loneliness (Appendix, Supplementary Table 4). Regarding the associations involving distinct ACE dimensions (Appendix, Supplementary Table 5), threat, loss, household dysfunction, and bonding were all associated with depressive symptoms; threat was associated with anxiety; and threat, household dysfunction, and bonding were associated with loneliness. These results suggest the threat dimension is more strongly related to psychological distress than other dimensions and further indicate the importance of looking at distinct types of ACEs to understand their specific effects on psychological distress. No changes were observed in the associations between CRP and psychological distress following exclusion of participants with CRP > 10 mg/L (Appendix, Supplementary Table 6). When using the continuous CRP variable (Appendix, Supplementary Table 7), the CRP-loneliness association was attenuated, whilst a new association and interaction between CRP and anxiety was observed. This questions the robustness of the CRP-loneliness association.

When using psychological distress at wave 9 as the outcome, hair cortisol and PGS were associated with greater levels of depression and anxiety (Appendix, Supplementary Table 8). ACEs were also associated with pre-pandemic psychological distress. Therefore, whilst PGS for depression and anxiety were not related to mental health responses to the pandemic, they were associated with the participants’ usual mental health before the pandemic. When using continuous depression and anxiety scores, whilst similar patterns of associations were observed overall, some discrepancies emerged. More specifically, cortisol was no longer associated with depression, whereas larger associations were observed between CRP and depression and between ACEs and anxiety during the pandemic (Appendix, Supplementary Table 9). No changes were observed when COVID-19-related worries were included as additional covariates in the mutually adjusted models for loneliness, depression, or anxiety (Appendix, Supplementary Table 10).

Following the false discovery rate adjustment, most associations remained significant. However, in the regression models, the association between ACEs and anxiety, and CRP and loneliness did not remain. Furthermore, the interactions between ACEs and hair cortisol only remained significant for anxiety, not for depression and loneliness. This suggests that these associations are weak and susceptible to multiple testing bias. False discovery rates are included in the tables for the main analyses (Tables 2–4). There were 4121 participants excluded from the current analyses since they did not take part in the life history interview or did not have biomarker/genetic data. The subgroup analyses comparing participants excluded and included in the current sample demonstrated that the two samples differed significantly by sex (<0.001), age (<0.001), partnership status (<0.001), education (<0.001), home tenure (<0.001), and presence of limiting long standing illness (0.015)(Appendix, Supplementary Table 11). For example, participants in the analytical sample were more likely to be women, be of older age, non-partnered, and own a property outright compared to those not included.

## Discussion

The current study identified social and biological risk markers for psychological distress among older adults during the COVID-19 pandemic. ACEs were consistently associated with all psychological distress outcomes, hair cortisol was associated with an increased risk of depression, and high CRP levels were related to greater loneliness scores. Interaction effects were also observed between ACEs and hair cortisol for all psychological distress outcomes, indicating that exposure to ACEs accentuated the relationship between hair cortisol and depression. Increases in psychological distress from before to during the pandemic were also observed for individuals with high CRP for loneliness, and multiple ACEs and higher hair cortisol levels for depression. PGS were not found to be predictive of their respective outcomes during the pandemic.

The first hypothesis stated that having greater ACEs, higher levels of cortisol and inflammation, and higher PGS would be associated with greater psychological distress during the pandemic. Several observed findings support this hypothesis. ACEs were found to be individually associated with all psychological distress outcomes during the pandemic, adjusting for pre-pandemic mental health. Furthermore, an increase in depressive symptoms from before to during the pandemic were observed specifically for individuals with multiple ACEs. These results suggest that the pandemic might have accentuated the psychological impact of ACEs. This supports previous literature observing increased psychopathology among individuals who had ACEs [6, 62]. Furthermore, it has been shown that childhood adversity can mould coping styles involved in developing healthy stress responses [63]. People affected by ACEs might therefore experience dysregulated stress perception, leading to a greater vulnerability to environmental stressors [64]. This greater susceptibility to stressors may in turn increase the risk of mental health problems [65]. Biological mechanisms may explain these adverse psychological reactions, including the impact of stress on physiological and neurological systems. Chronic stress from ACEs can cause physiological disruptions including HPA-axis dysregulation and heightened inflammation [15]. These impairments are in turn linked to impaired ability to adapt to new adversities [66], such as the pandemic.

The observed association between hair cortisol and depressive symptoms in the regression models adjusted for pre-pandemic mental health and the mixed effect models suggests that individuals with higher cortisol levels are at greater risk of negative psychological responses to the pandemic. A significant interaction effect with cortisol and wave was observed, but not a main effect of cortisol on depression. This suggests that cortisol seems to be more important in predicting the change in psychological distress compared to predicting the average level of depression across the timepoints. This is in line with existing literature as the pathophysiology of depression has been consistently associated with HPA-axis dysregulation [67]. These abnormalities are linked to a hyperactive stress response, including hypersensitivity to stressors and elevated body fluid cortisol concentrations [68]. A hyperactive stress response is in turn related to poorer mental health outcomes [65]. Of note, a recent meta-analysis demonstrated that different studies observe patients with depression to have increased, decreased, or no differences in hair cortisol concentrations compared to healthy individuals [69]. However, these inconsistencies are likely to be explained by differences in the study design, quality, and sample. Importantly, when using a continuous depression measure, the relationship with cortisol was attenuated. This suggests that this association may not be robust.

The observed association between CRP and loneliness in the regression models adjusted for pre-pandemic mental health and the mixed-effect models suggests that individuals with high pre-pandemic inflammation were particularly susceptible to the impact of the pandemic on loneliness. Systemic inflammation can induce behavioural, motivational, and perceptual changes including altered social behaviour and increased reactivity to threatening stimuli [70]. Therefore, participants with high CRP may have a heightened stress response and impaired social perceptions contributing to pandemic psychological distress, including feelings of loneliness. Research demonstrates that pre-trauma inflammation amplifies the negative effects of trauma on subsequent mental health [71]. This suggests interactions between inflammation and environmental stress on psychopathology. These effects may be explained by the impact of inflammation on the functional connectivity and activation of brain regions implicated in the pathophysiology of stress-related mental health outcomes, including loneliness [72], such as the pre-frontal cortex and amygdala [73]. Of note, when the continuous CRP measure was used, the association with loneliness was attenuated whilst a new association with anxiety was observed. This suggests that the findings may not be robust and should be interpreted with caution.

Whilst the above findings support the first hypothesis, not all the hypothesised associations were observed. No associations were observed between CRP and depression or anxiety. This contradicts a recent ELSA study that identified a relationship between pre-pandemic CRP and depression during the pandemic among older adults [74]. These discrepancies may be due to the current study having a smaller sample size and a larger time gap between the CRP and depression assessments. Furthermore, no associations between PGS and their respective psychological distress outcome during the pandemic were observed. Of note, PGS were found to predict pre-pandemic mental health in our sensitivity analysis. Another cohort study has found weak evidence for the association between PGS and depression and anxiety during the pandemic among young people, whereas PGS were related to psychological distress during the pandemic among middle-aged adults [75]. These results provide further insight into the gene-environment mechanisms influencing mental health. More specifically, they suggest that genetic factors linked to depression, anxiety, and loneliness may affect older adults’ usual mental health but may be less influential in their psychological reactions to new environmental stressors, and that the influence of genetic factors on an individual’s sensitivity to stressful circumstances may vary across different stages of the life course. Nevertheless, it is worth noting that associations between PGS and their respective mental health outcome are usually driven by small effect sizes. Considering the diminished sample size of this analysis due to inclusion criteria, a lack of power may explain the null associations reported here.

The second hypothesis stated that ACEs would interact with cortisol, inflammation, and PGS to further increase an individual’s risk of psychological distress. Support for this hypothesis comes from the observed interaction between ACEs and hair cortisol for psychological distress. This interaction suggests that their interplay might be instrumental in understanding the pandemic response among older adults. Individuals with HPA-axis hypersensitivity could be more susceptible to the negative psychological impact of ACEs. This supports the diathesis-stress model that postulates some individuals, due to their biology, are more vulnerable to adverse effects of negative experiences [76]. Alternatively, this interaction may suggest that childhood adversity is biologically embedded. Early adversity is linked to chronic secretion of glucocorticoids [77], which can hinder brain development [78] and lead to dysregulated stress reactivity [77]. Therefore, individuals exposed to ACEs may have had greater negative responses to the pandemic due to their biologically embedded heightened stress reactivity. As interactions between PGS and CRP with ACEs were not observed, it suggests that HPA related processes may be more relevant in the embedding of childhood adversity compared to inflammatory or genetic markers.

To our knowledge, this is the first study to prospectively investigate the impact of pre-existing social, biological, and genetic risk factors on older adults’ mental health responses to the pandemic. A key strength of this study is the large population-based sample providing more accurate mean estimates and improved generalisability. Nonetheless, the findings should be viewed in the context of several limitations. The sample lacked ethnic diversity which may be due to PGS being generated only from those from European descent or from selection biases. Considering the greater impact of the pandemic on ethnic minorities, this is an important demographic to include [79]. It is also recognised that the GWAS used to generate the anxiety PGS has now been superseded, but the newer PGS was not available when the analyses were conducted [80]. The current study used PGS with a single p-value threshold of 1, which may capture higher levels of genetic association, but could also increase noise and variance compared with lower p-value thresholds. Our sensitivity analyses demonstrate that the demographic characteristics of the analytical sample differed from those of ELSA participants excluded due to missing data on the exposures of interest, and such differences increase the likelihood of selection bias and question the generalisability of the findings. It is, however, important to acknowledge that some participants were not administered the relevant modules, including biological samples and ACEs, as they were part of refreshment samples from 2008, 2012, 2014, and 2016, meaning their missing data is not because they failed to provide responses.

The discrepancies observed within the sensitivity analyses using continuous CRP as an exposure and continuous depression and anxiety as outcomes suggest that the way the variables are handled may influence the observed associations. This is an important factor to consider when interpreting the current findings and in future research. Furthermore, the psychological distress measures and ACEs were self-reported which may introduce recall and social desirability biases. Recent studies also suggest that subjective, rather than objective, ACE accounts are associated with later psychopathology, which questions what the subjective measures reflect [81]. Similarly, the agreement between prospective and retrospective measures of childhood maltreatment has been questioned with a recent meta-analysis stating the importance of both acknowledging the critical measurement differences and not conflating the two concepts [50]. It is also possible that the observed associations reflect age-related changes rather than COVID-19-related changes. Finally, it is important to acknowledge the times at which the exposures were measured and how this may impact interpretation of findings. ACEs were assessed 14 years before the pandemic whilst CRP and hair cortisol were assessed 8 years before; different situational factors and mental health states may have impacted the assessment of exposure variables at different time points.

Nonetheless, the current study has important implications for older adult and childhood interventions by providing a more nuanced understanding of the aetiology of psychological distress. Firstly, it highlights the importance of providing support to reduce the adverse mental health impact of the pandemic among older adults, especially those with pre-existing risk factors. It also demonstrates a need for greater access to psychotherapeutic programmes to combat the impact of ACEs on later-life health [82], especially considering the likely increase in ACEs during the pandemic [83] and the fact that ACEs were a consistent predictor of psychological distress. These could include psychosocial interventions to manage the deleterious effects of ACEs and reduce stress levels or pharmacological therapies targeting biological pathways including the HPA-axis to improve symptoms. Finally, the findings are not only relevant in the context of the pandemic but can be translated more widely to understanding how individuals may react psychologically to new environmental stressors.

To conclude, this study suggests that individuals with ACEs are at greater risk of psychological distress during the COVID-19 pandemic. ACEs appear to be a more reliable marker of vulnerability compared to cortisol and CRP. However, dysregulated biomarkers of HPA-axis activity and inflammation pre-pandemic were also related to greater pandemic psychological distress. This implicates two biological pathways underlying psychological responses to new environmental stressors. Interactions between ACEs and hair cortisol on psychological distress during the pandemic were identified. Given the recognised role of the HPA-axis in the development of mental health conditions, the stress response system may characterise one of the main psychobiological mechanisms underlying the association of early adversity with poor adulthood mental health. Future longitudinal research that considers covariates including age, type and timing of adversity, different inflammatory markers (e.g., IL-6), and cortisol measurement, are necessary to understand the nuances of the biological pathways linking ACEs to psychopathology.

## Supplementary information

Supplementary material
