# Supplementary material for: The relationship of adverse childhood experiences, hair cortisol, C-reactive protein, and polygenic susceptibility with older adults’ psychological distress during the COVID-19 pandemic
Source: Mol Psychiatry. 2022 Oct 5;27(12):5038–48. doi: 10.1038/s41380-022-01805-2 (PMC9533280; doi:10.1038/s41380-022-01805-2)
Supplement: Supplementary file 1 — Supplementary material [file 41380_2022_1805_MOESM1_ESM.docx]

SUPPLEMENTARY MATERIALS

*C-reactive protein*

The exclusion criteria for blood sampling were clotting or bleeding disorders, history of fits or convulsions, and being on anticoagulant medication. Participants were asked to fast before their nurse visit unless they had diabetes and were on treatment or if they were unfit to fast. A minimum of three small tubes of blood (ranging from 2 ml to 6 ml in size) were collected from each participant. Following collection, the samples were frozen for long-term storage at the Royal Victoria Infirmary in Newcastle (UK). Plasma concentrations of CRP were assayed in duplicate from the 6 ml plain tubes using the N Latex CRP mono Immunoassay on the Behring Nephelometer II Analyser (Dade Behring, Milton Keynes, UK). The CRP values were expressed in mg/L. The coefficients of variation ranged between 3% and 7%. The reference range for CRP used by the laboratory was 0-5 mg/L. Values within this range were considered to be clinically ‘normal’, while those outside were treated as clinically ‘abnormal’. Further details about the blood sample analyses, internal quality control, and external quality assessment of the laboratory can be found in the ELSA Nurse Visit documentation(1).

*Polygenic risk scores (PGS)*

Using the Illumina HumanOmni2.5 BeadChips (HumanOmni2.5-4vl, HumanOmni2.5-8v1.3), University College London Genomics performed the genome-wide genotyping in ELSA between 2013-2014. Using PLINK, R, and VCFtools, SNPs were excluded if they were non-autosomal, the minor allele frequency was <0.01%, if more than 2% of genotype data were missing, and if the Hardy-Weinberg Equilibrium *p*-value was <10^-4^. Samples were removed based on call rate (<0.99), suspected non-European ancestry, sex difference in allelic frequency of ≥0.2, heterozygosity and relatedness. After quality control steps, 7 183 (96.9% n=7 412) individuals and 1372240 (61.5% of n=2230767 SNPs) directly genotyped SNPs remained for further analyses. To calculate PGSs for Major Depressive Disorder (MDD), anxiety, and loneliness, SNPs associated with each of these outcomes were weighted by their effect size derived from recent genome-wide meta-analyses of MDD, anxiety, and loneliness respectively, and summed in a continuous score using PRSice(2). As previous research highlighted that PGSs built from directly genotyped data either had more predictive power(3) or did not differ significantly from PGSs calculated using imputed data(4), we calculated PGSs based on genotyped data at different *P*-value cut-offs.

*Mixed effect models*

The current study used mixed effect models with random intercept, see the equation below:

$$\gamma_{ij}= \beta_{0}+\beta_{1}\chi_{ij}+\mathcal{u}_{j}+ \mathcal{e}_{ij}$$

This is a Random-Intercept only model: intercept ($\beta_{0}+\mathcal{u}_{j}$) varies across individuals; but the slope ($\beta_{1}$) does not vary across individuals.

Examples of the equations used in the current study:

- Loneliness = $\beta_{0}$ + ACEs*wave + covariates + $\mathcal{u}_{j}+ \mathcal{e}_{ij}$
- Loneliness = $\beta_{0}$ + CRP*wave + covariates + $\mathcal{u}_{j}+ \mathcal{e}_{ij}$
- Loneliness = $\beta_{0}$ + cortisol*wave + covariates + $\mathcal{u}_{j}+ \mathcal{e}_{ij}$
- Loneliness = $\beta_{0}$ + PGS*wave + covariates + $\mathcal{u}_{j}+ \mathcal{e}_{ij}$
- Depression = $\beta_{0}$ + ACEs*wave + covariates + $\mathcal{u}_{j}+ \mathcal{e}_{ij}$
- Depression = $\beta_{0}$ + CRP*wave + covariates + $\mathcal{u}_{j}+ \mathcal{e}_{ij}$
- Depression = $\beta_{0}$ + cortisol*wave + covariates + $\mathcal{u}_{j}+ \mathcal{e}_{ij}$
- Depression = $\beta_{0}$ + PGS*wave + covariates + $\mathcal{u}_{j}+ \mathcal{e}_{ij}$

The wave variable was a binary indicator of whether the psychological distress measurement was taken before the pandemic (Wave 9) or during the pandemic (COVID-19 assessment points 1 and 2) created when the dataset was transformed from wide to long format.

Supplementary Table 1: The number of participants that have individual ACEs (N = 2 050).

| Separation from mother |  |
| --- | --- |
| Missing | 2 |
| No | 1 776 (86.7%) |
| Yes | 272 (13.3%) |
| Physical abuse |  |
| Missing | 242 |
| No | 1 742 (96.3%) |
| Yes | 66 (3.7%) |
| Sexual abuse |  |
| No | 1 942 (94.7%) |
| Yes | 108 (5.3%) |
| Physical attack |  |
| Missing | 244 |
| No | 1 758 (97.3%) |
| Yes | 48 (2.7%) |
| Parent arguments |  |
| Missing | 258 |
| No | 1 360 (75.9%) |
| Yes | 432 (24.1%) |
| Institutionalisation |  |
| No | 2 024 (98.7%) |
| Yes | 26 (1.3%) |
| Foster care or adoption |  |
| No | 2 012 (98.1%) |
| Yes | 38 (1.9%) |
| Parent death |  |
| Missing | 2 |
| No | 1 962 (95.8%) |
| Yes | 86 (4.2%) |
| Poor maternal bonding |  |
| Missing | 346 |
| No | 1 370 (80.4%) |
| Yes | 334 (19.6%) |
| Poor paternal bonding |  |
| Missing | 380 |
| No | 1 324 (79.3%) |
| Yes | 346 (20.7%) |
| Parent mental illness or substance abuse | |
| Missing | 248 |
| No | 1 658 (92.0%) |
| Yes | 144 (8.0%) |

Supplementary Table 2: Dimensions of Adverse Childhood Experiences (ACEs)

| Dimension | Related ACEs |
| --- | --- |
| Threat | Sexual abuse, physical abuse from parents, physical assault |
| Household dysfunction | Parent arguments, parent mental illness or substance abuse, divorce or separation |
| Low parental bonding | Maternal bonding, paternal bonding |
| Loss | Parent death, maternal separation, foster care or adoption, institutionalisation |

Supplementary Table 3: Regression models of all interactions between total ACEs and either hair cortisol, CRP, or PGS for depression, anxiety, and loneliness.

| **Term** | **Model** | **Estimate**  **(OR/b)** | **Standard error** | **P value** | **Confidence low** | **Confidence high** |
| --- | --- | --- | --- | --- | --- | --- |
| **Depression** |  |  |  |  |  |  |
| Total ACEs | 1 | 1.054 | 0.091 | 0.566 | 0.878 | 1.258 |
| Hair cortisol | 1 | 1.060 | 0.065 | 0.369 | 0.931 | 1.200 |
| High CRP | 1 | 1.035 | 0.179 | 0.847 | 0.725 | 1.464 |
| MDD PGS | 1 | 1.060 | 0.081 | 0.468 | 0.906 | 1.244 |
| ACEs*Cortisol | 1 | 1.069 | 0.034 | 0.048 | 1.001 | 1.142 |
| Total ACEs | 2 | 1.267 | 0.063 | <0.001 | 1.118 | 1.430 |
| High CRP | 2 | 1.182 | 0.222 | 0.451 | 0.760 | 1.816 |
| Hair cortisol | 2 | 1.144 | 0.050 | 0.007 | 1.036 | 1.261 |
| MDD PGS | 2 | 1.064 | 0.080 | 0.439 | 0.910 | 1.247 |
| ACEs*High CRP | 2 | 0.913 | 0.099 | 0.358 | 0.750 | 1.109 |
| MDD PGS | 3 | 1.135 | 0.101 | 0.209 | 0.934 | 1.386 |
| Total ACEs | 3 | 1.250 | 0.053 | <0.001 | 1.124 | 1.386 |
| High CRP | 3 | 1.050 | 0.178 | 0.782 | 0.737 | 1.483 |
| Hair cortisol | 3 | 1.145 | 0.050 | 0.007 | 1.036 | 1.262 |
| MDD PGS*ACEs | 3 | 0.944 | 0.054 | 0.281 | 0.849 | 1.049 |
| **Anxiety** |  |  |  |  |  |  |
| Total ACEs | 1 | 0.814 | 0.134 | 0.123 | 0.621 | 1.050 |
| High CRP | 1 | 1.088 | 0.253 | 0.738 | 0.656 | 1.771 |
| ANX PGS | 1 | 0.917 | 0.115 | 0.452 | 0.732 | 1.148 |
| ACEs*Cortisol | 1 | 1.166 | 0.049 | 0.002 | 1.060 | 1.283 |
| Total ACEs | 1 | 1.119 | 0.087 | 0.192 | 0.939 | 1.320 |
| High CRP | 2 | 1.049 | 0.324 | 0.883 | 0.549 | 1.958 |
| Hair cortisol | 2 | 1.082 | 0.076 | 0.301 | 0.929 | 1.252 |
| ANX PGS | 2 | 0.906 | 0.113 | 0.385 | 0.725 | 1.132 |
| ACEs*High CRP | 2 | 1.051 | 0.137 | 0.716 | 0.800 | 1.370 |
| Total ACEs | 2 | 1.140 | 0.070 | 0.061 | 0.989 | 1.301 |
| ANX PGS | 3 | 0.972 | 0.143 | 0.840 | 0.735 | 1.288 |
| High CRP | 3 | 1.151 | 0.249 | 0.572 | 0.699 | 1.863 |
| Hair cortisol | 3 | 1.081 | 0.076 | 0.307 | 0.927 | 1.251 |
| ANX PGS*ACEs | 3 | 0.947 | 0.068 | 0.429 | 0.824 | 1.078 |
| **Loneliness** |  |  |  |  |  |  |
| Hair cortisol | 1 | -0.043 | 0.024 | 0.074 | -0.090 | 0.004 |
| Total ACEs | 1 | 0.015 | 0.039 | 0.701 | -0.061 | 0.090 |
| High CRP | 1 | 0.147 | 0.071 | 0.038 | 0.008 | 0.285 |
| LONE PGS | 1 | -0.030 | 0.030 | 0.312 | -0.089 | 0.028 |
| ACEs*Cortisol | 1 | 0.039 | 0.015 | 0.012 | 0.008 | 0.069 |
| Total ACEs | 2 | 0.092 | 0.027 | 0.001 | 0.040 | 0.144 |
| High CRP | 2 | 0.142 | 0.087 | 0.103 | -0.029 | 0.313 |
| Hair cortisol | 2 | -0.009 | 0.020 | 0.666 | -0.047 | 0.030 |
| LONE PGS | 2 | -0.032 | 0.030 | 0.278 | -0.091 | 0.026 |
| ACEs*High CRP | 2 | 0.006 | 0.046 | 0.899 | -0.085 | 0.096 |
| Total ACEs | 3 | 0.097 | 0.023 | <0.001 | 0.053 | 0.141 |
| LONE PGS | 3 | -0.019 | 0.037 | 0.600 | -0.092 | 0.053 |
| High CRP | 3 | 0.150 | 0.071 | 0.034 | 0.011 | 0.289 |
| Hair cortisol | 3 | -0.009 | 0.020 | 0.653 | -0.048 | 0.030 |
| LONE PGS*ACEs | 3 | -0.013 | 0.021 | 0.545 | -0.053 | 0.028 |

^1^ Having 0 ACEs and not having high CRP were the reference categories.

^2^ Odds ratios are presented for anxiety and depression.

^3^ MDD PGS, ANX PGS, and LONE PGS are the PGS for depression, anxiety, and loneliness, respectively.

^4^ Model 1 represents the mutually adjusted model including an interaction term between total ACEs and hair cortisol; Model 2 represents the mutually adjusted model including an interaction term between total ACEs and CRP; Model 3 represents the mutually adjusted model including an interaction term between total ACEs and PGS.

^5^ Covariates included in the models: pre-pandemic psychological distress (depression, anxiety, and loneliness), adult sociodemographic factors (age, gender, partnership status, living alone, wealth tertiles), use of medication (anti-inflammatory/antihypertensive drugs, steroids), childhood socioeconomic factors (overcrowding, number of books in home, financial hardship, parent unemployment), hair characteristics (dyed, colour, season of collection, phase of hair analysis), and genetic population stratification by including 10 principal components.

Supplementary Table 4: Regression models of the mutually adjusted associations of ACEs, hair cortisol, CRP, and PGS with depression, anxiety, and loneliness adjusted for lifestyle factors and limiting long standing illness.

| **Term** | **Estimate**  **(OR/b)** | **Standard error** | **P value** |
| --- | --- | --- | --- |
| **Depression** |  |  |  |
| 1 ACE | 2.153 | 0.210 | 0.000 |
| 2+ ACEs | 2.516 | 0.199 | <0.001 |
| Hair cortisol | 1.182 | 0.054 | 0.002 |
| High CRP | 0.882 | 0.207 | 0.543 |
| MDD PGS | 1.046 | 0.089 | 0.615 |
| **Anxiety** |  |  |  |
| 1 ACE | 2.608 | 0.310 | 0.002 |
| 2+ ACEs | 1.866 | 0.286 | 0.029 |
| Hair cortisol | 1.128 | 0.082 | 0.142 |
| High CRP | 1.124 | 0.280 | 0.677 |
| ANX PGS | 0.890 | 0.121 | 0.336 |
| **Loneliness** |  |  |  |
| 1 ACE | -0.066 | 0.077 | 0.388 |
| 2+ ACEs | 0.318 | 0.076 | <0.001 |
| Hair cortisol | -0.009 | 0.021 | 0.657 |
| High CRP | 0.139 | 0.077 | 0.072 |
| LONE PGS | -0.028 | 0.031 | 0.372 |

^1^ Having 0 ACEs and not having high CRP were the reference categories.

^2^ Odds ratios are presented for anxiety and depression.

^3^ MDD PGS, ANX PGS, and LONE PGS are the PGS, anxiety, and loneliness, respectively.

^4^ Covariates included in the models: lifestyle factors (BMI, alcohol consumption, physical activity, smoking), limiting longstanding illness, pre-pandemic psychological distress (depression, anxiety, and loneliness), adult sociodemographic factors (age, gender, partnership status, living alone, wealth tertiles), use of medication (anti-inflammatory/antihypertensive drugs, steroids), childhood socioeconomic factors (overcrowding, number of books in home, financial hardship, parent unemployment), hair characteristics (dyed, colour, season of collection, phase of hair analysis), and genetic population stratification by including 10 principal components.

Supplementary Table 5: Regression models of the individual associations between distinct ACE dimensions and depression, anxiety, and loneliness.

| **Term** | **Estimate**  **(OR/b)** | **Standard error** | **P value** |
| --- | --- | --- | --- |
| **Depression** |  |  |  |
| Threat | 1.592 | 0.230 | 0.043 |
| Loss | 1.475 | 0.189 | 0.040 |
| Household dysfunction | 1.889 | 0.159 | <0.001 |
| Bonding | 1.553 | 0.168 | 0.009 |
| **Anxiety** |  |  |  |
| Threat | 2.130 | 0.275 | 0.006 |
| Loss | 1.134 | 0.279 | 0.653 |
| Household dysfunction | 1.077 | 0.229 | 0.746 |
| Bonding | 1.038 | 0.237 | 0.874 |
| **Loneliness** |  |  |  |
| Threat | 0.291 | 0.105 | 0.006 |
| Loss | 0.035 | 0.082 | 0.673 |
| Household dysfunction | 0.215 | 0.068 | 0.002 |
| Bonding | 0.240 | 0.069 | <0.001 |

^1^ Odds ratios are presented for anxiety and depression.

^2^ Covariates included in the models: pre-pandemic psychological distress (depression, anxiety, and loneliness), adult sociodemographic factors (age, gender, partnership status, living alone, wealth tertiles), use of medication (anti-inflammatory/antihypertensive drugs, steroids), and childhood socioeconomic factors (overcrowding, number of books in home, financial hardship, parent unemployment).

Supplementary Table 6: Regression models of the mutually adjusted associations of ACEs, hair cortisol, CRP, and PGS with depression, anxiety, and loneliness excluding CRP >10mg/l.

| **Term** | **Estimate**  **(OR/b)** | **Standard error** | **P value** |
| --- | --- | --- | --- |
| **Depression** |  |  |  |
| Total ACEs | 1.221 | 0.051 | <0.001 |
| Hair cortisol | 1.104 | 0.053 | 0.063 |
| High CRP | 0.990 | 0.196 | 0.961 |
| MDD PGS | 1.022 | 0.083 | 0.791 |
| Total ACEs | 1.009 | 0.093 | 0.922 |
| Hair cortisol | 0.993 | 0.070 | 0.924 |
| High CRP | 0.975 | 0.198 | 0.900 |
| MDD PGS | 1.015 | 0.084 | 0.861 |
| ACEs*Cortisol | 1.091 | 0.035 | 0.012 |
| Total ACEs | 1.263 | 0.063 | <0.001 |
| High CRP | 1.144 | 0.247 | 0.586 |
| Hair cortisol | 1.105 | 0.053 | 0.060 |
| MDD PGS | 1.021 | 0.083 | 0.804 |
| ACEs*High CRP | 0.905 | 0.106 | 0.349 |
| MDD PGS | 1.097 | 0.104 | 0.376 |
| Total ACEs | 1.247 | 0.054 | <0.001 |
| High CRP | 0.996 | 0.197 | 0.982 |
| Hair cortisol | 1.106 | 0.053 | 0.058 |
| MDD PGS*ACEs | 0.940 | 0.054 | 0.252 |
| **Anxiety** |  |  |  |
| Total ACEs | 1.181 | 0.070 | 0.018 |
| Hair cortisol | 1.087 | 0.081 | 0.305 |
| High CRP | 0.999 | 0.276 | 0.998 |
| ANX PGS | 0.938 | 0.120 | 0.595 |
| Hair cortisol | 0.873 | 0.115 | 0.239 |
| Total ACEs | 0.869 | 0.131 | 0.284 |
| High CRP | 0.969 | 0.281 | 0.909 |
| ANX PGS | 0.950 | 0.122 | 0.672 |
| ACEs*Cortisol | 1.157 | 0.049 | 0.003 |
| Total ACEs | 1.098 | 0.088 | 0.287 |
| High CRP | 0.708 | 0.371 | 0.352 |
| Hair cortisol | 1.085 | 0.081 | 0.319 |
| ANX PGS | 0.933 | 0.120 | 0.563 |
| ACEs*High CRP | 1.231 | 0.142 | 0.145 |
| Total ACEs | 1.188 | 0.072 | 0.017 |
| ANX PGS | 1.065 | 0.155 | 0.683 |
| High CRP | 1.038 | 0.277 | 0.893 |
| Hair cortisol | 1.081 | 0.082 | 0.339 |
| ANX PGS*ACEs | 0.912 | 0.071 | 0.196 |
| **Loneliness** |  |  |  |
| Total ACEs | 0.096 | 0.022 | <0.001 |
| Hair cortisol | -0.011 | 0.020 | 0.600 |
| High CRP | 0.177 | 0.076 | 0.020 |
| LONE PGS | -0.024 | 0.030 | 0.432 |
| Hair cortisol | -0.049 | 0.024 | 0.045 |
| Total ACEs | 0.010 | 0.039 | 0.804 |
| High CRP | 0.175 | 0.076 | 0.021 |
| LONE PGS | -0.022 | 0.030 | 0.468 |
| ACEs*Cortisol | 0.042 | 0.015 | 0.006 |
| Total ACEs | 0.092 | 0.026 | 0.001 |
| High CRP | 0.158 | 0.094 | 0.092 |
| Hair cortisol | -0.010 | 0.020 | 0.602 |
| LONE PGS | -0.024 | 0.030 | 0.432 |
| ACEs*High CRP | 0.017 | 0.048 | 0.730 |
| Total ACEs | 0.100 | 0.023 | <0.001 |
| LONE PGS | -0.008 | 0.038 | 0.829 |
| High CRP | 0.178 | 0.076 | 0.019 |
| Hair cortisol | -0.011 | 0.020 | 0.590 |
| LONE PGS*ACEs | -0.015 | 0.021 | 0.473 |

^1^ Not having high CRP was the reference category.

^2^ Odds ratios are presented for anxiety and depression.

^3^ MDD PGS, ANX PGS, and LONE PGS are the PGS for depression, anxiety, and loneliness, respectively.

^4^ Covariates included in the models: pre-pandemic psychological distress (depression, anxiety, and loneliness), adult sociodemographic factors (age, gender, partnership status, living alone, wealth tertiles), use of medication (anti-inflammatory/antihypertensive drugs, steroids), childhood socioeconomic factors (overcrowding, number of books in home, financial hardship, parent unemployment), hair characteristics (dyed, colour, season of collection, phase of hair analysis), and genetic population stratification by including 10 principal components.

Supplementary Table 7: Regression models of the mutually adjusted associations of ACEs, hair cortisol, continuous CRP, and PGS with depression, anxiety, and loneliness.

| **Term** | **Estimate**  **(OR/b)** | **Standard error** | **P value** |
| --- | --- | --- | --- |
| **Depression** |  |  |  |
| Total ACEs | 1.224 | 0.050 | <0.001 |
| Hair cortisol | 1.144 | 0.050 | 0.007 |
| CRP | 1.000 | 0.011 | 0.995 |
| MDD PGS | 1.064 | 0.081 | 0.441 |
| Total ACEs | 1.054 | 0.091 | 0.564 |
| Hair cortisol | 1.060 | 0.065 | 0.364 |
| CRP | 1.000 | 0.011 | 0.976 |
| MDD PGS | 1.060 | 0.081 | 0.472 |
| ACEs*Cortisol | 1.069 | 0.034 | 0.047 |
| Total ACEs | 1.264 | 0.055 | <0.001 |
| CRP | 1.011 | 0.014 | 0.407 |
| Hair cortisol | 1.143 | 0.050 | 0.008 |
| MDD PGS | 1.065 | 0.081 | 0.431 |
| ACEs*CRP | 0.993 | 0.006 | 0.209 |
| MDD PGS | 1.134 | 0.101 | 0.213 |
| Total ACEs | 1.251 | 0.053 | <0.001 |
| CRP | 1.000 | 0.011 | 0.966 |
| Hair cortisol | 1.146 | 0.050 | 0.007 |
| MDD PGS*ACEs | 0.944 | 0.054 | 0.284 |
| **Anxiety** |  |  |  |
| Total ACEs | 1.143 | 0.069 | 0.052 |
| Hair cortisol | 1.085 | 0.076 | 0.285 |
| CRP | 1.002 | 0.020 | 0.919 |
| ANX PGS | 0.904 | 0.113 | 0.375 |
| Hair cortisol | 0.872 | 0.107 | 0.203 |
| Total ACEs | 0.813 | 0.134 | 0.122 |
| CRP | 1.001 | 0.020 | 0.955 |
| ANX PGS | 0.915 | 0.115 | 0.440 |
| ACEs*Cortisol | 1.168 | 0.048 | 0.001 |
| Total ACEs | 1.248 | 0.075 | 0.003 |
| CRP | 1.038 | 0.019 | 0.047 |
| Hair cortisol | 1.088 | 0.076 | 0.267 |
| ANX PGS | 0.887 | 0.113 | 0.289 |
| ACEs*CRP | 0.983 | 0.007 | 0.014 |
| Total ACEs | 1.142 | 0.070 | 0.058 |
| ANX PGS | 0.964 | 0.142 | 0.797 |
| CRP | 1.001 | 0.020 | 0.945 |
| Hair cortisol | 1.083 | 0.076 | 0.296 |
| ANX PGS*ACEs | 0.951 | 0.068 | 0.459 |
| **Loneliness** |  |  |  |
| Total ACEs | 0.097 | 0.022 | <0.001 |
| Hair cortisol | -0.006 | 0.020 | 0.752 |
| CRP | 0.001 | 0.004 | 0.893 |
| LONE PGS | -0.033 | 0.030 | 0.269 |
| Hair cortisol | -0.041 | 0.024 | 0.088 |
| Total ACEs | 0.017 | 0.039 | 0.665 |
| CRP | 0.001 | 0.004 | 0.874 |
| LONE PGS | -0.031 | 0.030 | 0.303 |
| ACEs*Cortisol | 0.039 | 0.015 | 0.011 |
| Total ACEs | 0.108 | 0.024 | <0.001 |
| CRP | 0.005 | 0.006 | 0.403 |
| Hair cortisol | -0.006 | 0.020 | 0.745 |
| LONE PGS | -0.032 | 0.030 | 0.281 |
| ACEs*CRP | -0.003 | 0.003 | 0.265 |
| Total ACEs | 0.100 | 0.023 | <0.001 |
| LONE PGS | -0.021 | 0.037 | 0.572 |
| CRP | 0.001 | 0.004 | 0.878 |
| Hair cortisol | -0.007 | 0.020 | 0.740 |
| LONE PGS*ACEs | -0.012 | 0.021 | 0.574 |

^1^ Odds ratios are presented for anxiety and depression.

^2^ MDD PGS, ANX PGS, and LONE PGS are the PGS for depression, anxiety, and loneliness, respectively.

^3^ Covariates included in the models: pre-pandemic psychological distress (depression, anxiety, and loneliness), adult sociodemographic factors (age, gender, partnership status, living alone, wealth tertiles), use of medication (anti-inflammatory/antihypertensive drugs, steroids), childhood socioeconomic factors (overcrowding, number of books in home, financial hardship, parent unemployment), hair characteristics (dyed, colour, season of collection, phase of hair analysis), and genetic population stratification by including 10 principal components.

Supplementary Table 8: Regression models of individual associations of ACEs, hair cortisol, CRP, and PGS with pre-pandemic depression, anxiety, and loneliness.

| **Term** | **Estimate**  **(OR/b)** | **Standard Error** | **P value** | **Confidence low** | **Confidence high** |
| --- | --- | --- | --- | --- | --- |
| **Depression** |  |  |  |  |  |
| 1 ACE | 1.260 | 0.219 | 0.293 | 0.813 | 1.920 |
| 2+ ACEs | 1.490 | 0.198 | 0.043 | 1.010 | 2.190 |
| Hair cortisol | 0.845 | 0.064 | 0.009 | 0.742 | 0.955 |
| High CRP | 1.160 | 0.189 | 0.437 | 0.793 | 1.670 |
| MDD PGS | 1.325 | 0.087 | 0.001 | 1.119 | 1.573 |
| **Anxiety** |  |  |  |  |  |
| 1 ACE | 0.197 | 0.144 | 0.173 | -0.086 | 0.480 |
| 2+ ACEs | 0.725 | 0.140 | <0.001 | 0.451 | 0.999 |
| Hair cortisol | -0.095 | 0.038 | 0.013 | -0.171 | -0.020 |
| High CRP | -0.062 | 0.136 | 0.650 | -0.328 | 0.205 |
| ANX PGS | -0.001 | 0.001 | 0.024 | -0.002 | 0.000 |
| **Loneliness** |  |  |  |  |  |
| 1 ACE | 0.290 | 0.099 | 0.004 | 0.095 | 0.484 |
| 2+ ACEs | 0.472 | 0.096 | <0.001 | 0.283 | 0.661 |
| Hair cortisol | -0.022 | 0.027 | 0.416 | -0.074 | 0.030 |
| High CRP | -0.151 | 0.093 | 0.105 | -0.333 | 0.031 |
| LONE PGS | 0.075 | 0.041 | 0.067 | -0.005 | 0.155 |

^1^ Having 0 ACEs and not having high CRP were the reference categories.

^2^ Odds ratios are presented for depression.

^3^ The pre-pandemic anxiety measure was the Office for National Statistics anxiety scale, which is a continuous measure.

^4^ MDD PGS, ANX PGS, and LONE PGS are the PGS for depression, anxiety, and loneliness, respectively.

^5^ Covariates included in the models: pre-pandemic psychological distress (depression, anxiety, and loneliness), adult sociodemographic factors (age, gender, partnership status, living alone, wealth tertiles), use of medication (anti-inflammatory/antihypertensive drugs, steroids), childhood socioeconomic factors (overcrowding, number of books in home, financial hardship, parent unemployment), hair characteristics (dyed, colour, season of collection, phase of hair analysis), and genetic population stratification by including 10 principal components.

Supplementary Table 9: Mutually adjusted regression models of ACEs, hair cortisol, CRP, and PGS with continuous depression and anxiety outcome measures.

| Term | Estimate (b) | Standard Error | P value | Confidence low | Confidence high |
| --- | --- | --- | --- | --- | --- |
| **Depression** |  |  |  |  |  |
| Total ACEs | 0.153 | 0.025 | 0.000 | 0.104 | 0.202 |
| Hair cortisol | 0.030 | 0.023 | 0.179 | -0.014 | 0.075 |
| High CRP | 0.241 | 0.080 | 0.003 | 0.084 | 0.399 |
| MDD PGS | 0.041 | 0.035 | 0.230 | -0.026 | 0.109 |
| **Anxiety** |  |  |  |  |  |
| Total ACEs | 0.238 | 0.053 | 0.000 | 0.134 | 0.342 |
| Hair cortisol | 0.035 | 0.048 | 0.462 | -0.059 | 0.129 |
| High CRP | 0.209 | 0.173 | 0.225 | -0.129 | 0.548 |
| ANX PGS | 0.002 | 0.073 | 0.976 | -0.141 | 0.145 |

^1^ Not having high CRP was the reference category.

^2^ MDD PGS, ANX PGS, and LONE PGS are the PGS for depression, anxiety, and loneliness, respectively.

^3^ Covariates included in the models: pre-pandemic psychological distress (depression, anxiety, and loneliness), adult sociodemographic factors (age, gender, partnership status, living alone, wealth tertiles), use of medication (anti-inflammatory/antihypertensive drugs, steroids), childhood socioeconomic factors (overcrowding, number of books in home, financial hardship, parent unemployment), hair characteristics (dyed, colour, season of collection, phase of hair analysis), and genetic population stratification by including 10 principal components.

Supplementary Table 10: Regression models of the mutually adjusted associations of ACEs, hair cortisol, CRP, and PGS with depression, anxiety, and loneliness adjusted for COVID-19-related worries.

| Term | Estimate (OR/b) | Standard Error | P value | Confidence low | Confidence high |
| --- | --- | --- | --- | --- | --- |
| **Loneliness** |  |  |  |  |  |
| 1 ACE | -0.029 | 0.075 | 0.697 | -0.175 | 0.117 |
| 2+ ACEs | 0.285 | 0.073 | 0.000 | 0.141 | 0.428 |
| Hair cortisol | -0.012 | 0.020 | 0.557 | -0.050 | 0.027 |
| High CRP | 0.131 | 0.071 | 0.064 | -0.008 | 0.270 |
| LONE PGS | -0.032 | 0.030 | 0.277 | -0.091 | 0.026 |
| **Depression** |  |  |  |  |  |
| 1 ACE | 2.051 | 0.203 | 0.000 | 1.376 | 3.050 |
| 2+ ACEs | 2.663 | 0.189 | 0.000 | 1.840 | 3.868 |
| Hair cortisol | 1.126 | 0.051 | 0.021 | 1.017 | 1.243 |
| High CRP | 0.984 | 0.183 | 0.931 | 0.684 | 1.403 |
| MDD PGS | 1.067 | 0.083 | 0.434 | 0.908 | 1.256 |
| **Anxiety** |  |  |  |  |  |
| 1 ACE | 1.893 | 0.300 | 0.033 | 1.046 | 3.399 |
| 2+ ACEs | 1.862 | 0.272 | 0.022 | 1.093 | 3.186 |
| Hair cortisol | 1.081 | 0.077 | 0.312 | 0.926 | 1.254 |
| High CRP | 0.925 | 0.267 | 0.770 | 0.541 | 1.546 |
| ANX PGS | 0.877 | 0.119 | 0.272 | 0.694 | 1.108 |

^1^ Not having high CRP was the reference category.

^2^ Odds ratios are presented for anxiety and depression.

^3^ MDD PGS, ANX PGS, and LONE PGS are the PGS for depression, anxiety, and loneliness, respectively.

^4^ Covariates included in the models: COVID-19-related worries (financial situation worse than before the pandemic, participant contacted by the NHS or GP and advised that they are vulnerable and at risk of severe illness if you catch coronavirus, friend or family member died with coronavirus, whether participant has had COVID), pre-pandemic psychological distress (depression, anxiety, and loneliness), adult sociodemographic factors (age, gender, partnership status, living alone, wealth tertiles), use of medication (anti-inflammatory/antihypertensive drugs, steroids), childhood socioeconomic factors (overcrowding, number of books in home, financial hardship, parent unemployment), hair characteristics (dyed, colour, season of collection, phase of hair analysis), and genetic population stratification by including 10 principal components.

Supplementary Table 11: Comparison of the characteristics of the analytical sample versus excluded participants at the first COVID-19 assessment.

|  | **Excluded (N=4 121)** | **Included (N=2 050)** | **p value** |
| --- | --- | --- | --- |
| **Sex** |  |  | < 0.001 |
| Male | 1 864 (45.2%) | 702 (34.2%) |  |
| Female | 2 257 (54.8%) | 1 348 (65.8%) |  |
| **Age** |  |  | < 0.001 |
| Mean (SD) | 68.588 (9.348) | 75.146 (6.505) |  |
| **Partnership** |  |  | < 0.001 |
| Partnered | 3 093 (75.1%) | 1 432 (69.9%) |  |
| Non-partnered | 1 028 (24.9%) | 618 (30.1%) |  |
| **Education** |  |  | < 0.001 |
| High | 1 003 (24.5%) | 416 (20.3%) |  |
| Medium | 1 982 (48.4%) | 1 002 (48.9%) |  |
| Low | 1 114 (27.2%) | 632 (30.8%) |  |
| **Home tenure** |  |  | < 0.001 |
| Owns outright | 2 921 (71.0%) | 1 766 (86.1%) |  |
| Owns with mortgage | 691 (16.8%) | 78 (3.8%) |  |
| Rents | 503 (12.2%) | 206 (10.0%) |  |
| **Wealth (numerical score)** |  |  | 0.279 |
| Mean (SD) | 215 280.847  (525 084.328) | 230 104.904  (465 169.266) |  |
| **Limiting longstanding illness** |  |  | 0.015 |
| Yes | 2 193 (53.2%) | 1 158 (56.5%) |  |
| No | 1 928 (46.8%) | 892 (43.5%) |  |
